# Supplementary material for: Single-cell transcriptome analysis indicates fatty acid metabolism-mediated metastasis and immunosuppression in male breast cancer
Source: Nat Commun. 2023 Sep 11;14:5590. doi: 10.1038/s41467-023-41318-2 (PMC10495415; doi:10.1038/s41467-023-41318-2)
Supplement: Supplementary file 1 — Supplementary Information [file 41467_2023_41318_MOESM1_ESM.pdf]

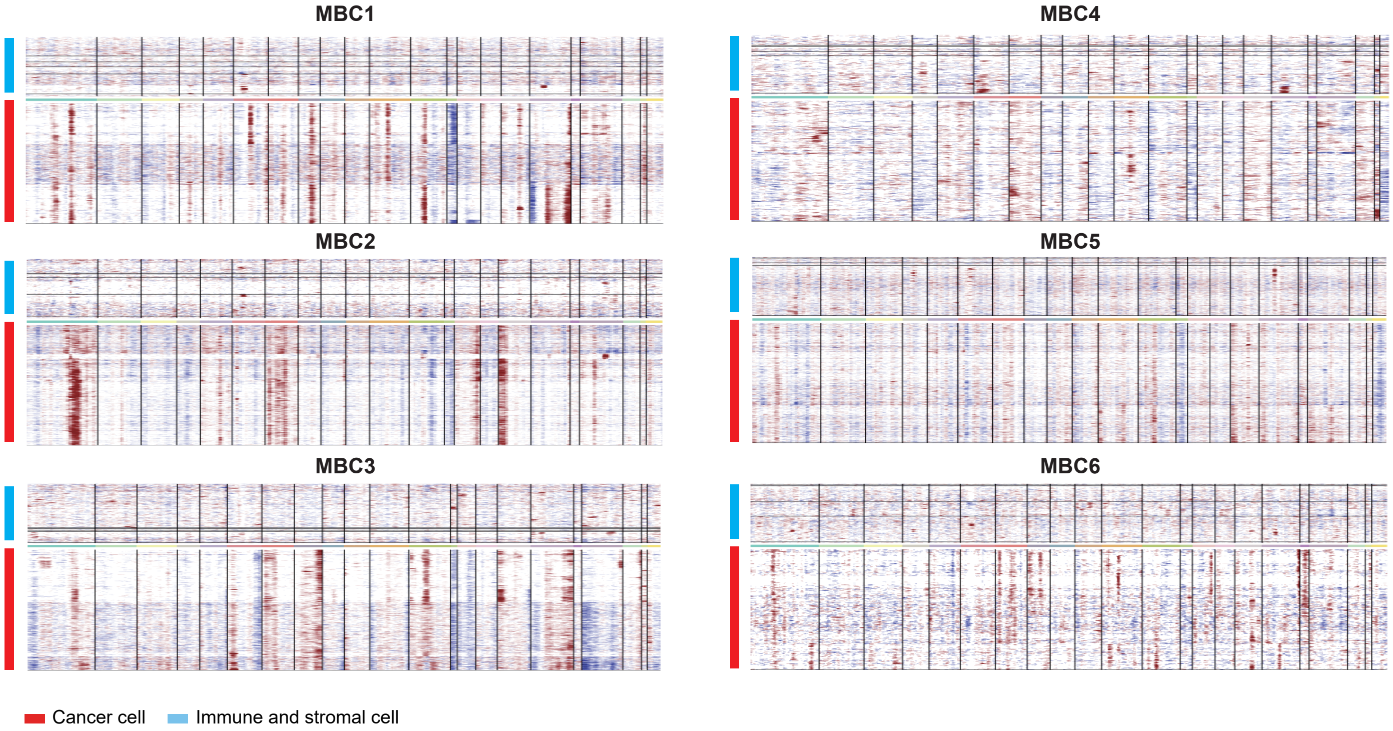

Supplementary Figure 1. The chromosomal landscape of inferred large-scale CNVs distinguishing malignant cells from non-malignant cells of MBC samples. Red color represents amplifications, and blue color represents deletions.

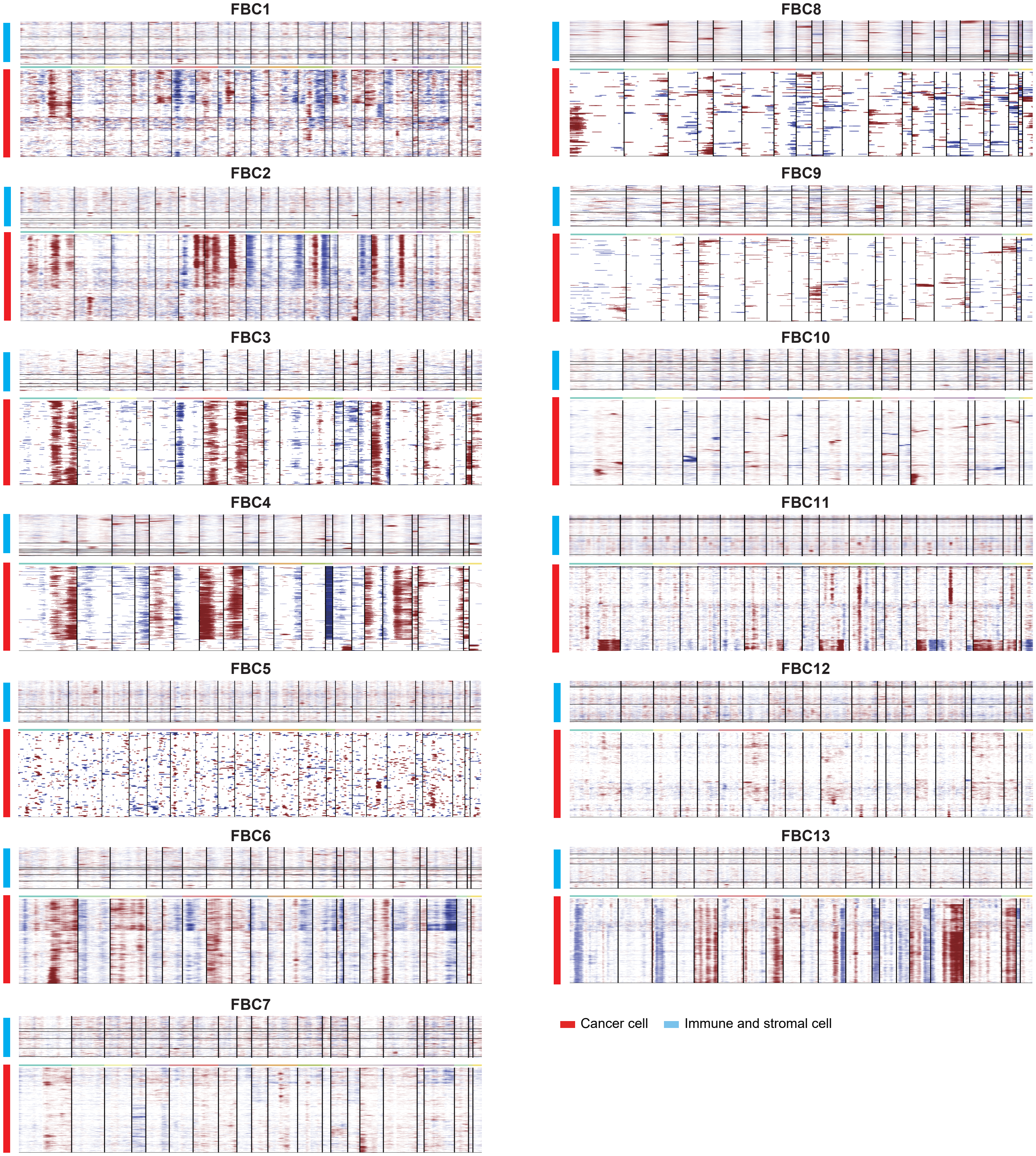

Supplementary Figure 2. The chromosomal landscape of inferred large-scale CNVs distinguishing malignant cells from non-malignant cells of FBC samples. Red color represents amplifications, and blue color represents deletions.

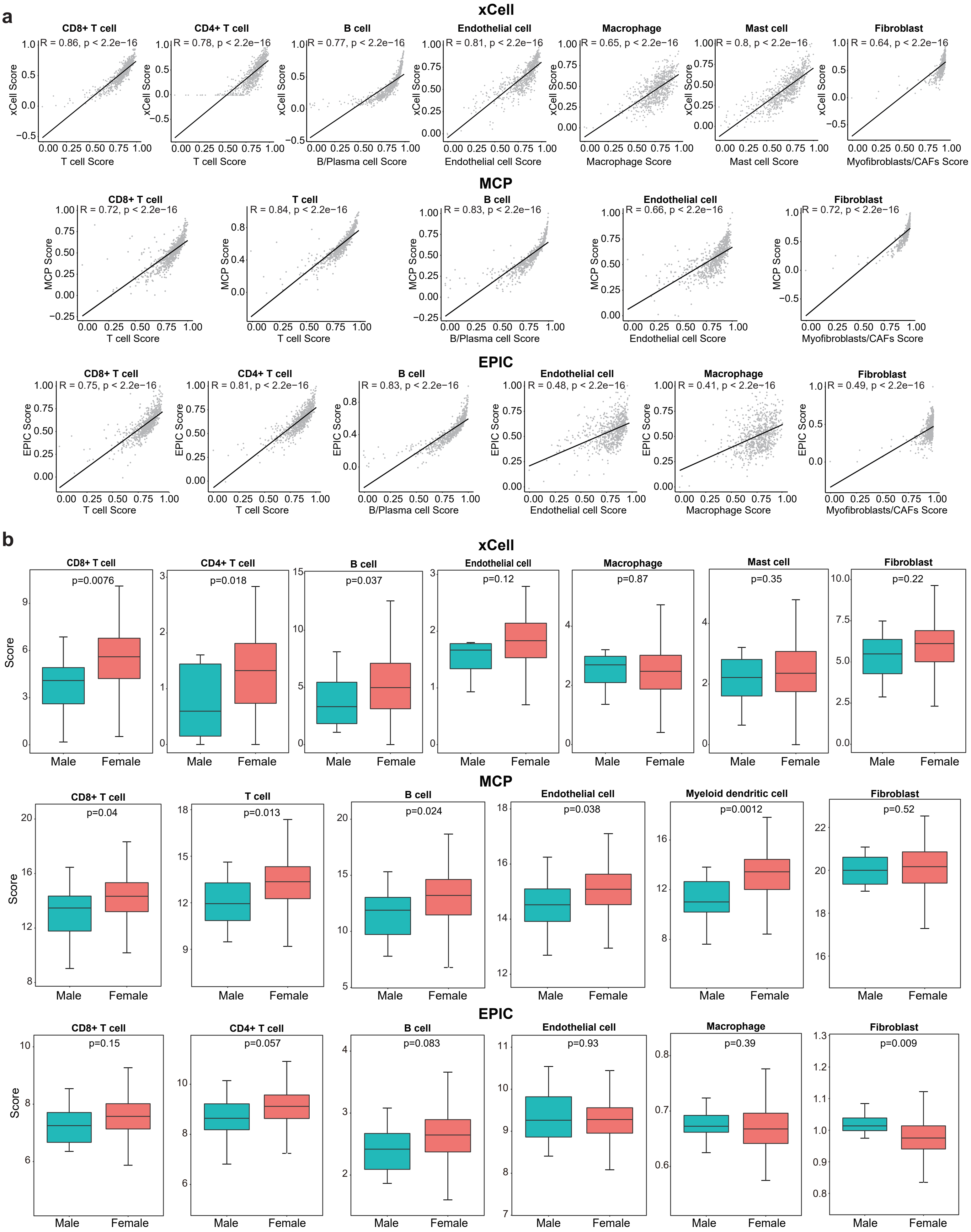

a
xCell
CD8+ T cell
R = 0.86, p < 2.2e−16
CD4+ T cell
R = 0.78, p < 2.2e−16
B cell
R = 0.77, p < 2.2e−16
Endothelial cell
R = 0.81, p < 2.2e−16
Macrophage
R = 0.65, p < 2.2e−16
Mast cell
R = 0.8, p < 2.2e−16
Fibroblast
R = 0.64, p < 2.2e−16
xCell Score
T cell Score
B/Plasma cell Score
Endothelial cell Score
Macrophage Score
Mast cell Score
Myofibroblasts/CAFs Score
MCP
CD8+ T cell
R = 0.72, p < 2.2e−16
T cell
R = 0.84, p < 2.2e−16
B cell
R = 0.83, p < 2.2e−16
Endothelial cell
R = 0.66, p < 2.2e−16
Fibroblast
R = 0.72, p < 2.2e−16
MCP Score
EPIC
CD8+ T cell
R = 0.75, p < 2.2e−16
CD4+ T cell
R = 0.81, p < 2.2e−16
B cell
R = 0.83, p < 2.2e−16
Endothelial cell
R = 0.48, p < 2.2e−16
Macrophage
R = 0.41, p < 2.2e−16
Fibroblast
R = 0.49, p < 2.2e−16
EPIC Score
b
xCell
CD8+ T cell p=0.0076
CD4+ T cell p=0.018
B cell p=0.037
Endothelial cell p=0.12
Macrophage p=0.87
Mast cell p=0.35
Fibroblast p=0.22
MCP
CD8+ T cell p=0.04
T cell p=0.013
B cell p=0.024
Endothelial cell p=0.038
Myeloid dendritic cell p=0.0012
Fibroblast p=0.52
EPIC
CD8+ T cell p=0.15
CD4+ T cell p=0.057
B cell p=0.083
Endothelial cell p=0.93
Macrophage p=0.39
Fibroblast p=0.009
Score
Male
Female

Supplementary Figure 3. Cellular components in TCGA MBC and FBC ER+ samples inferred by immune-deconvolution tools. **a** The Pearson correlation analysis of putative cell type levels derived from single-cell signatures and immune-deconvolution tools. **b** Boxplot showing the scores of immune and stromal cells in TCGA MBC (n = 12) and FBC ER+ (n = 710) samples inferred by xCell, MCP, and EPIC. P-value was calculated by two-sided Wilcoxon rank-sum test. Box plots show median (center line), the upper and lower quantiles (box), and the range of the data (whiskers).

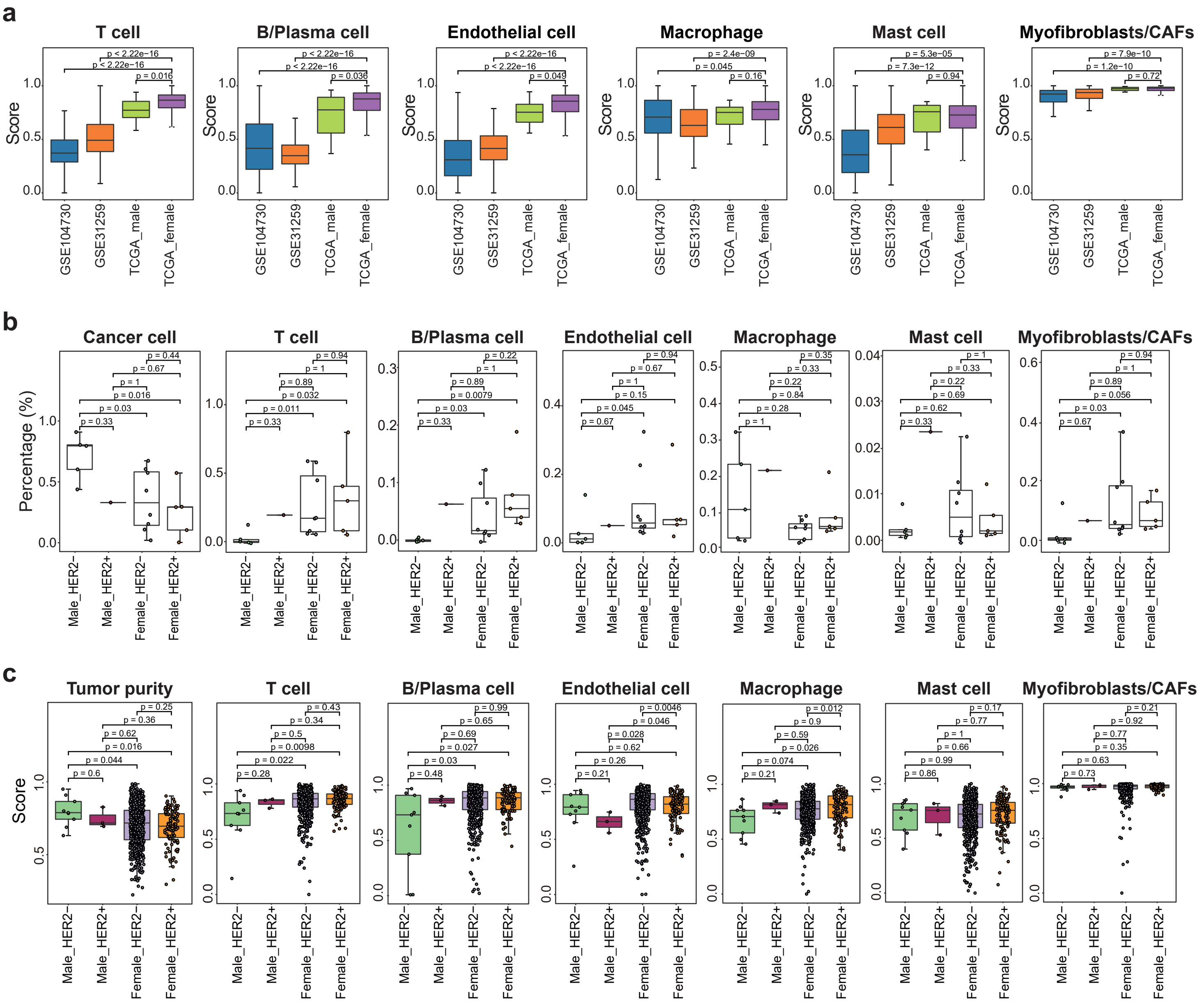

Supplementary Figure 4. Comparison of cellular components between MBC and FBC in independent datasets. a Boxplots showing the signature scores of T cells, B/Plasma cells, endothelial cells, macrophages, mast cells and myofibroblast/CAFs in ER+ MBC samples from GSE104730 (n =46), GSE31259 (n = 74), and TCGA (n = 12) datasets, as well as ER+ FBC (n = 710) samples from TCGA dataset. P-value was calculated by two-sided Wilcoxon rank-sum test. **b** Boxplot showing the percentage of cancer cells, T cells, B/Plasma cells, endothelial cells, macrophages, mast cells and myofibroblast/CAFs in ER+HER2- MBC (n = 5), ER+HER2+ MBC (n = 1), ER+HER2- FBC (n = 8), and ER+HER2+ FBC (n = 5) samples in scRNA-seq data. HER2 status is defined by IHC experiments. **c** Boxplot showing the tumor purity and signature scores of T cells, B/Plasma cells, endothelial cells, macrophages, mast cells and myofibroblast/CAFs in ER+HER2- MBC (n = 9), ER+HER2+ MBC (n = 3), ER+HER2- FBC (n = 598), and ER+HER2+ FBC (n = 112) in TCGA ER+ BRCA cohort. HER2 status is based on the IHC results in the clinical information of TCGA-BRCA dataset. P-value was calculated by two-sided Wilcoxon rank-sum test. In (**a-c**), box plots show median (center line), the upper and lower quantiles (box), and the range of the data (whiskers). Source data are provided as a Source Data file.

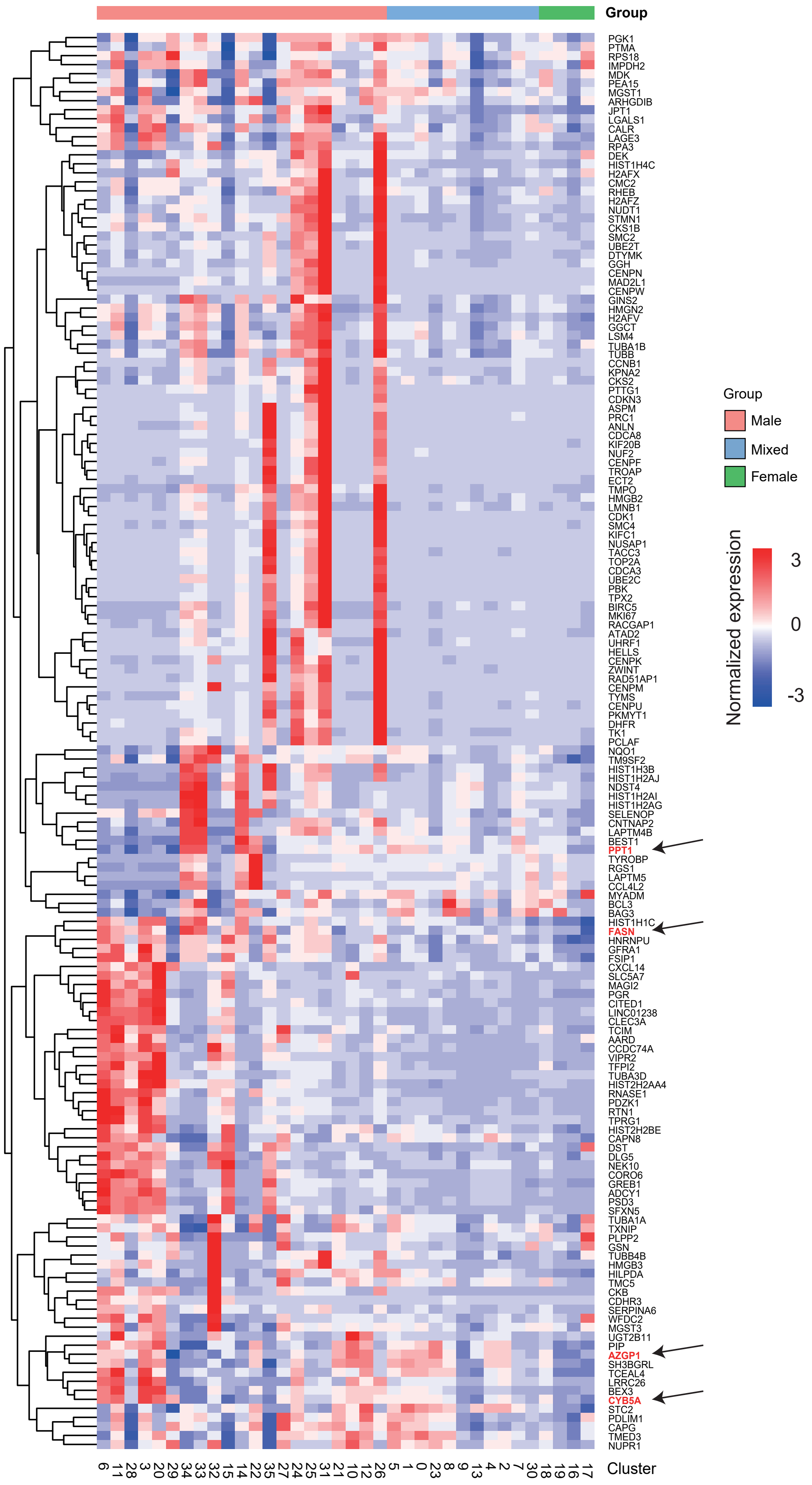
Group
PGK1
PTMA
RPS18
IMPDH2
MDK
PEA15
MGST1
ARHGDIB
JPT1
LGALS1
CALR
LAGE3
RPA3
DEK
HIST1H4C
H2AFX
CMC2
RHEB
H2AFZ
NUDT1
STMN1
CKS1B
SMC2
UBE2T
DTYMK
GGH
CENPN
MAD2L1
CENPW
GINS2
HMGN2
H2AFV
GGCT
LSM4
TUBA1B
TUBB
CCNB1
KPNA2
CKS2
PTTG1
CDKN3
ASPM
PRC1
ANLN
CDCA8
KIF20B
NUF2
CENPF
TROAP
ECT2
TMPO
HMGB2
LMNB1
CDK1
SMC4
KIFC1
NUSAP1
TACC3
TOP2A
CDCA3
UBE2C
PBK
TPX2
BIRC5
MKI67
RACGAP1
ATAD2
UHRF1
HELLS
CENPK
ZWINT
RAD51AP1
CENPM
TYMS
CENPU
PKMYT1
DHFR
TK1
PCLAF
NQO1
TM9SF2
HIST1H3B
HIST1H2AJ
NDST4
HIST1H2AI
HIST1H2AG
SELENOP
CNTNAP2
LAPTM4B
BEST1
PPT1
TYROBP
RGS1
LAPTM5
CCL4L2
MYADM
BCL3
BAG3
HIST1H1C
FASN
HNRNPU
GFRA1
FSIP1
CXCL14
SLC5A7
MAGI2
PGR
CITED1
LINC01238
CLEC3A
TCIM
AARD
CCDC74A
VIPR2
TFPI2
TUBA3D
HIST2H2AA4
RNASE1
PDZK1
RTN1
TPRG1
HIST2H2BE
CAPN8
DST
DLG5
NEK10
CORO6
GREB1
ADCY1
PSD3
SFXN5
TUBA1A
TXNIP
PLPP2
GSN
TUBB4B
HMGB3
HILPDA
TMC5
CKB
CDHR3
SERPINA6
WFDC2
MGST3
UGT2B11
PIP
AZGP1
SH3BGRL
TCEAL4
LRRC26
BEX3
CYB5A
STC2
PDLIM1
CAPG
TMED3
NUPR1
6 11 28 3 20 29 34 33 32 15 14 22 35 27 24 25 31 21 10 12 26 5 1 0 23 8 9 13 4 2 7 30 18 19 16 17
Cluster
Group
Male
Mixed
Female
Normalized expression
3
0
-3

Supplementary Figure 5. The expression levels of specifically expressed genes of male cancer cell clusters. Genes with log2 fold change greater than 0.25 and adjusted p-value less than 0.01 for each cluster were identified using the MAST method with default parameters. Gene markers that presented in at least three male clusters (lightcoral) were selected, and markers of female (turquoise) or mixed (lightblue) clusters were further removed from this list. Scale bar depicting low expression in blue and high expression in red.

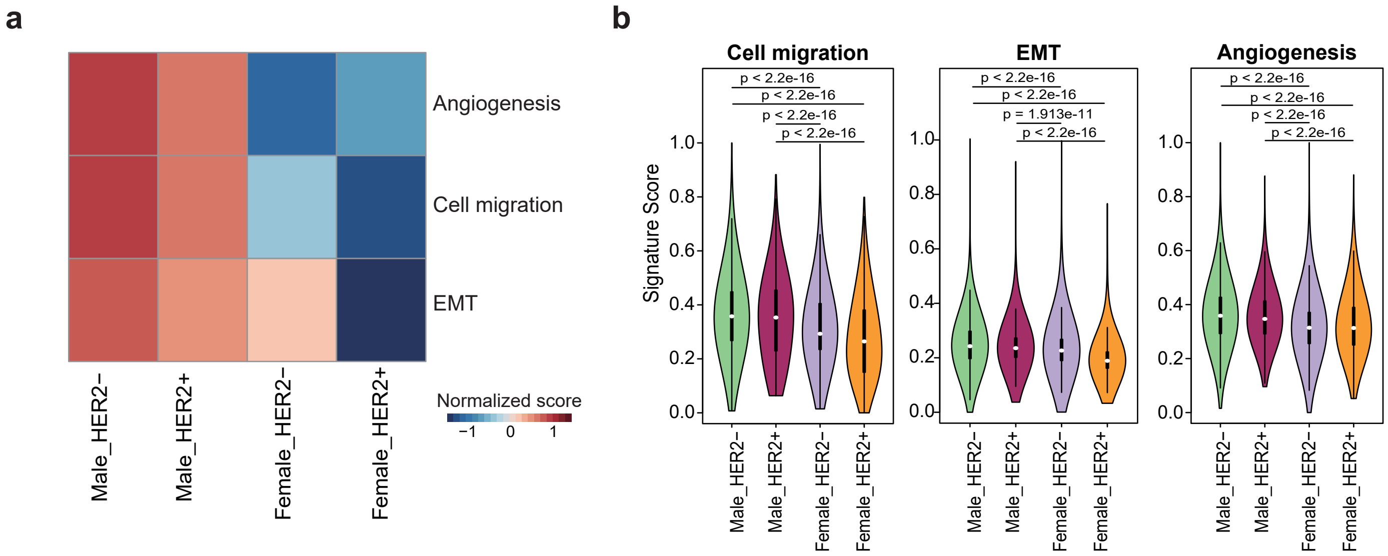

Supplementary Figure 6. Comparison of metastasis signature scores of cancer cells in ER+HER2- MBC, ER+HER2+ MBC, ER+HER2- FBC, and ER+HER2+ FBC samples. a Heatmap showing the average ssGSEA scores of cell migration, EMT and angiogenesis in cancer cells from ER+HER2- MBC, ER+HER2+ MBC, ER+HER2- FBC, and ER+HER2+ FBC samples. Scale bar depicting low score in blue and high score in red. **b** Violin plots comparing the scores of cell migration, EMT and angiogenesis of cancer cells from ER+HER2- MBC (n = 34815 cells), ER+HER2+ MBC (n = 2455 cells), ER+HER2- FBC (n = 12563 cells), and ER+HER2+ FBC (n = 3510 cells) samples. P-value was calculated by two-sided Wilcoxon rank-sum test. Box plots show median (center line), the upper and lower quantiles (box), and the range of the data (whiskers). Source data are provided as a Source Data file.

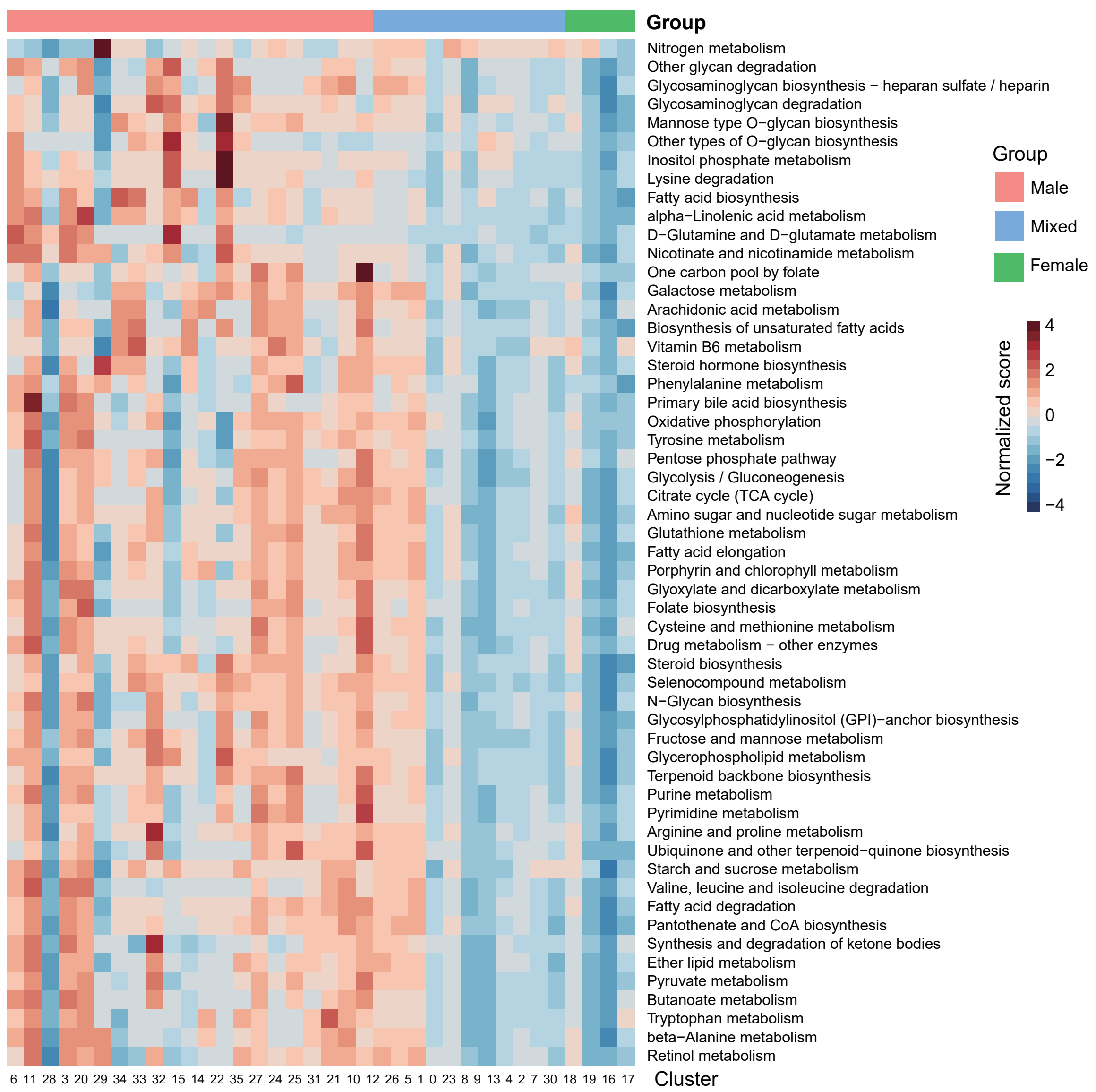

Supplementary Figure 7. The differentially activated metabolism pathways between male and mixed/female cancer cell clusters. Pathways with adjusted p-value less than 0.05 were shown. Scale bar depicting low score in blue and high score in red. Lightcoral represents male clusters, turquoise represents female clusters and lightblue represents mixed clusters.

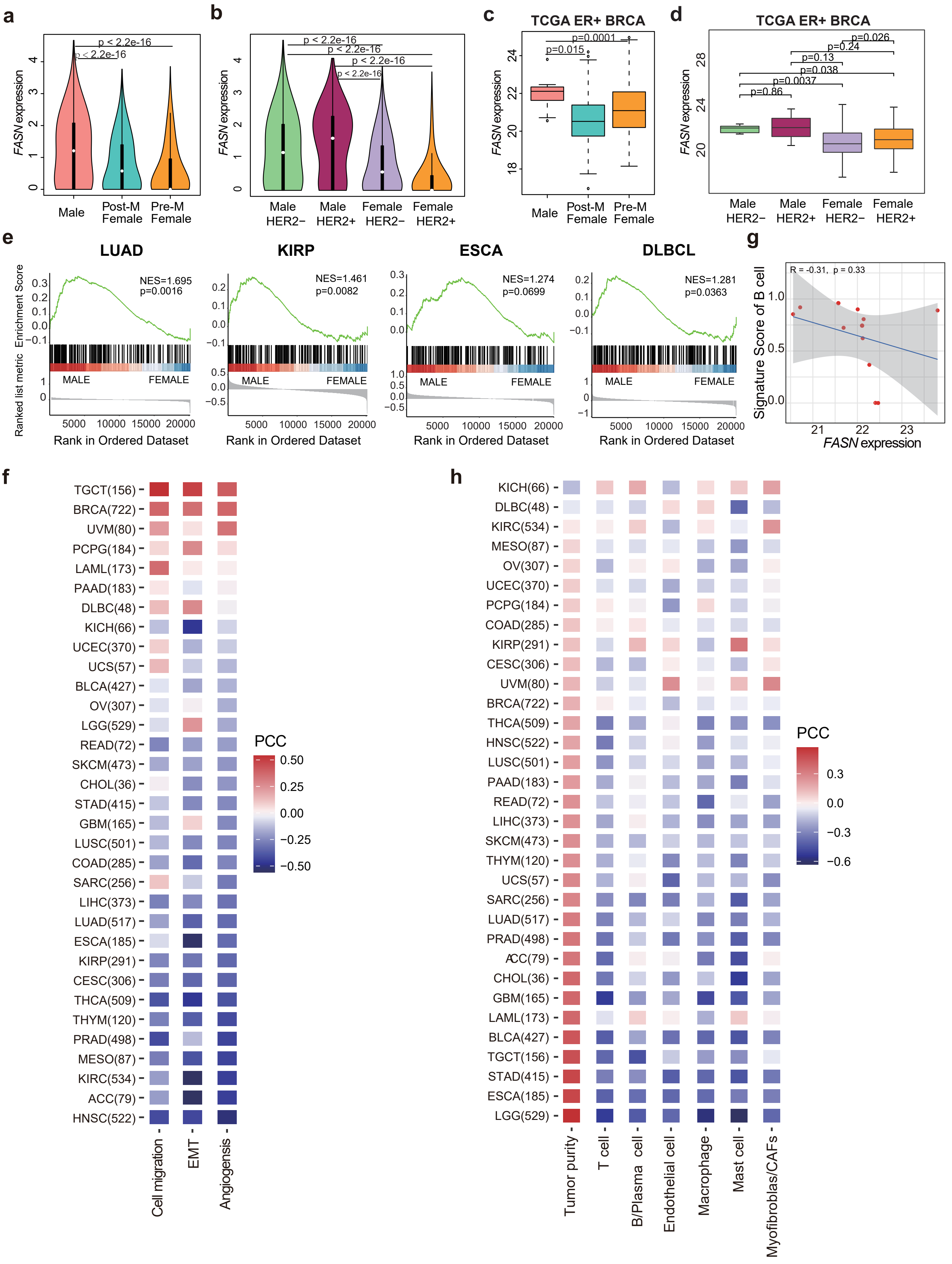

a
p < 2.2e-16
p < 2.2e-16
FASN expression
Male
Post-M Female
Pre-M Female
b
p < 2.2e-16
p < 2.2e-16
p < 2.2e-16
p < 2.2e-16
FASN expression
Male HER2−
Male HER2+
Female HER2−
Female HER2+
c
TCGA ER+ BRCA
p=0.0001
p=0.015
FASN expression
Male
Post-M Female
Pre-M Female
d
TCGA ER+ BRCA
p=0.026
p=0.24
p=0.13
p=0.038
p=0.0037
p=0.86
FASN expression
Male HER2−
Male HER2+
Female HER2−
Female HER2+
e
LUAD
NES=1.695
p=0.0016
KIRP
NES=1.461
p=0.0082
ESCA
NES=1.274
p=0.0699
DLBCL
NES=1.281
p=0.0363
Enrichment Score
Ranked list metric
MALE
FEMALE
Rank in Ordered Dataset
g
R = -0.31, p = 0.33
Signature Score of B cell
FASN expression
f
TGCT(156)
BRCA(722)
UVM(80)
PCPG(184)
LAML(173)
PAAD(183)
DLBC(48)
KICH(66)
UCEC(370)
UCS(57)
BLCA(427)
OV(307)
LGG(529)
READ(72)
SKCM(473)
CHOL(36)
STAD(415)
GBM(165)
LUSC(501)
COAD(285)
SARC(256)
LIHC(373)
LUAD(517)
ESCA(185)
KIRP(291)
CESC(306)
THCA(509)
THYM(120)
PRAD(498)
MESO(87)
KIRC(534)
ACC(79)
HNSC(522)
Cell migration
EMT
Angiogensis
PCC
h
KICH(66)
DLBC(48)
KIRC(534)
MESO(87)
OV(307)
UCEC(370)
PCPG(184)
COAD(285)
KIRP(291)
CESC(306)
UVM(80)
BRCA(722)
THCA(509)
HNSC(522)
LUSC(501)
PAAD(183)
READ(72)
LIHC(373)
SKCM(473)
THYM(120)
UCS(57)
SARC(256)
LUAD(517)
PRAD(498)
ACC(79)
CHOL(36)
GBM(165)
LAML(173)
BLCA(427)
TGCT(156)
STAD(415)
ESCA(185)
LGG(529)
Tumor purity
T cell
B/Plasma cell
Endothelial cell
Macrophage
Mast cell
Myofibroblas/CAFs
PCC

Supplementary Figure 8. The role of fatty acid metabolism in various cancer types. a Violin plot showing *FASN* expression in cancer cells of MBC (n = 37270 cells), postmenopausal (n = 8437 cells) and premenopausal (n = 7636 cells) FBC samples in scRNA-seq dataset. P-value was calculated by two-sided Wilcoxon rank-sum test. **b** Violin plot showing *FASN* expression in cancer cells from ER+HER2- MBC (n = 34815 cells), ER+HER2+ MBC (n = 2455 cells), ER+HER2- FBC (n = 12563 cells), and ER+HER2+ FBC (n = 3510 cells) samples in scRNA-seq dataset. P-value was calculated by two-sided Wilcoxon rank-sum test. **c** Boxplot showing the *FASN* expression among male (n = 12), postmenopausal (n = 337) and premenopausal (n = 372) female samples in TCGA ER+ BRCA cohort. P-value was calculated by two-sided Wilcoxon rank-sum test. **d** Boxplot showing the *FASN* expression among ER+HER2- MBC (n = 9), ER+HER2+ MBC (n = 3), ER+HER2- FBC (n = 598), and ER+HER2+ FBC (n = 112) samples in TCGA BRCA cohort. P-value was calculated by two-sided Wilcoxon rank-sum test. **e** GSEA analysis of fatty acid metabolism pathway between male and female patients in various cancer types. LUAD: Lung adenocarcinoma; KIRP: Kidney renal papillary cell carcinoma; ESCA: Esophageal carcinoma; DLBCL: Lymphoid Neoplasm Diffuse Large B-cell Lymphoma; NES: normalized enrichment score. Two-sided kolmogorov–smirnov test was used to calculate p-value. **f** Heatmap showing the Pearson correlation coefficient between the signature score of fatty acid metabolism and metastasis-related programs in the various cancer types of TCGA cohort. Red color represents positive correlation, and blue color represents negative correlation. **g** The Pearson correlation analysis between *FASN* expression and the signature score of B cells for MBC samples in TCGA ER+ BRCA cohort. 95% confidence interval (CI) is indicated with gray color. **h** Heatmap showing the Pearson correlation coefficient between expression of *FASN* and tumor purity, immune and stromal cells scores in various cancer types of TCGA cohort. Red color represents positive correlation, and blue color represents negative correlation. In (**a-d**), box plots show median (center line), the upper and lower quantiles (box), and the range of the data (whiskers). Source data are provided as a Source Data file.

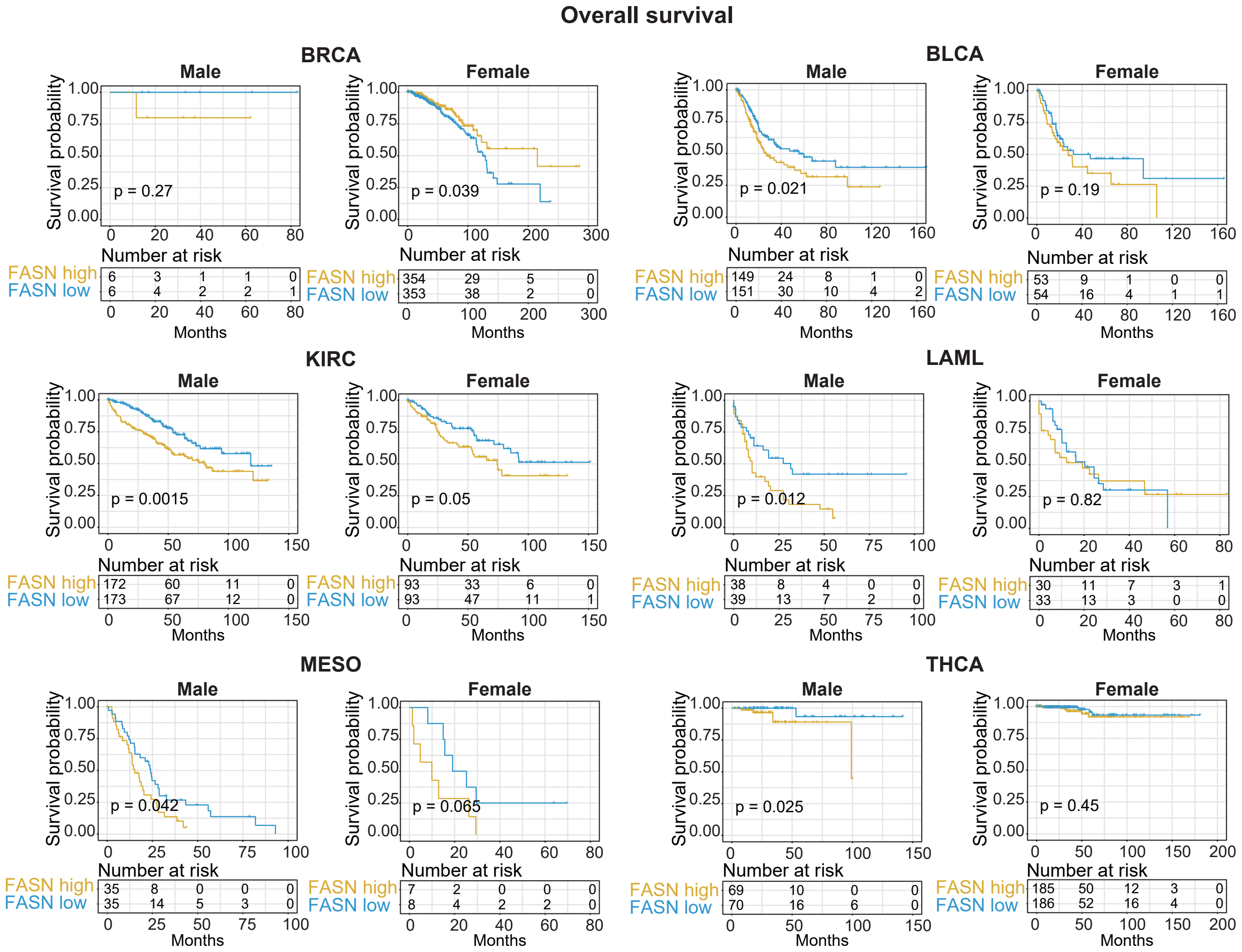

Supplementary Figure 9. Overall survival analysis of male and female patients in various cancer types based on the *FASN* expression. Patients are categorized into *FASN*-high (yellow) and *FASN*-low (blue) groups for each dataset according to the median of *FASN* expression. The significance was evaluated by the log-rank test. BRCA: Breast invasive carcinoma; BLCA: Bladder Urothelial Carcinoma; KIRC: Kidney renal clear cell carcinoma; LAML: Acute Myeloid Leukemia; MESO: Mesothelioma; THCA: Thyroid carcinoma.

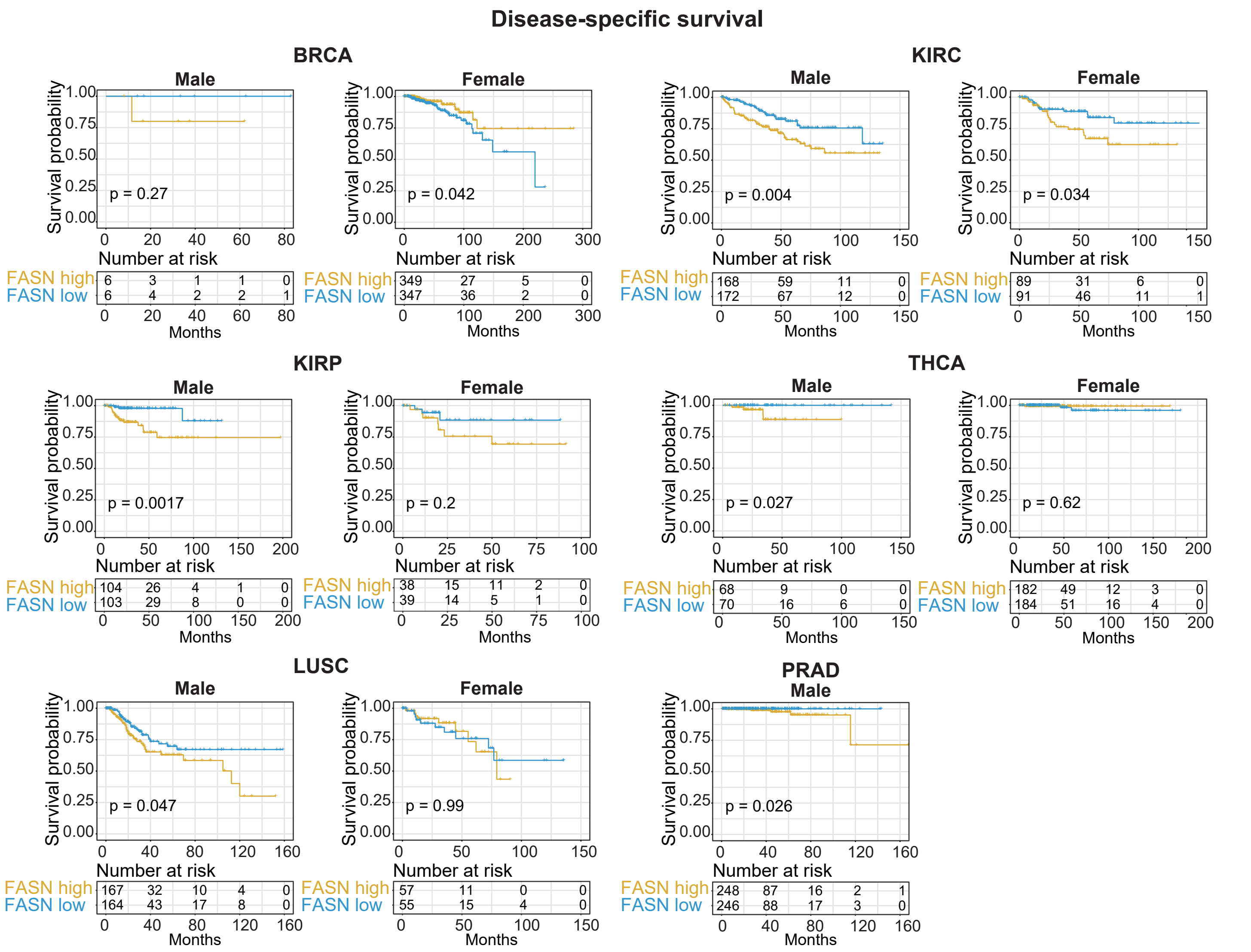

Supplementary Figure 10. Disease-specific survival analysis of male and female patients in various cancer types based on the *FASN* expression. Patients are categorized into *FASN*-high (yellow) and *FASN*-low (blue) groups for each dataset according to the median of *FASN* expression. The significance was evaluated by the log-rank test. BRCA: Breast invasive carcinoma; KIRC: Kidney renal clear cell carcinoma; KIRP: Kidney renal papillary cell carcinoma; THCA: Thyroid carcinoma; LUSC: Lung squamous cell carcinoma; PRAD: Prostate adenocarcinoma.

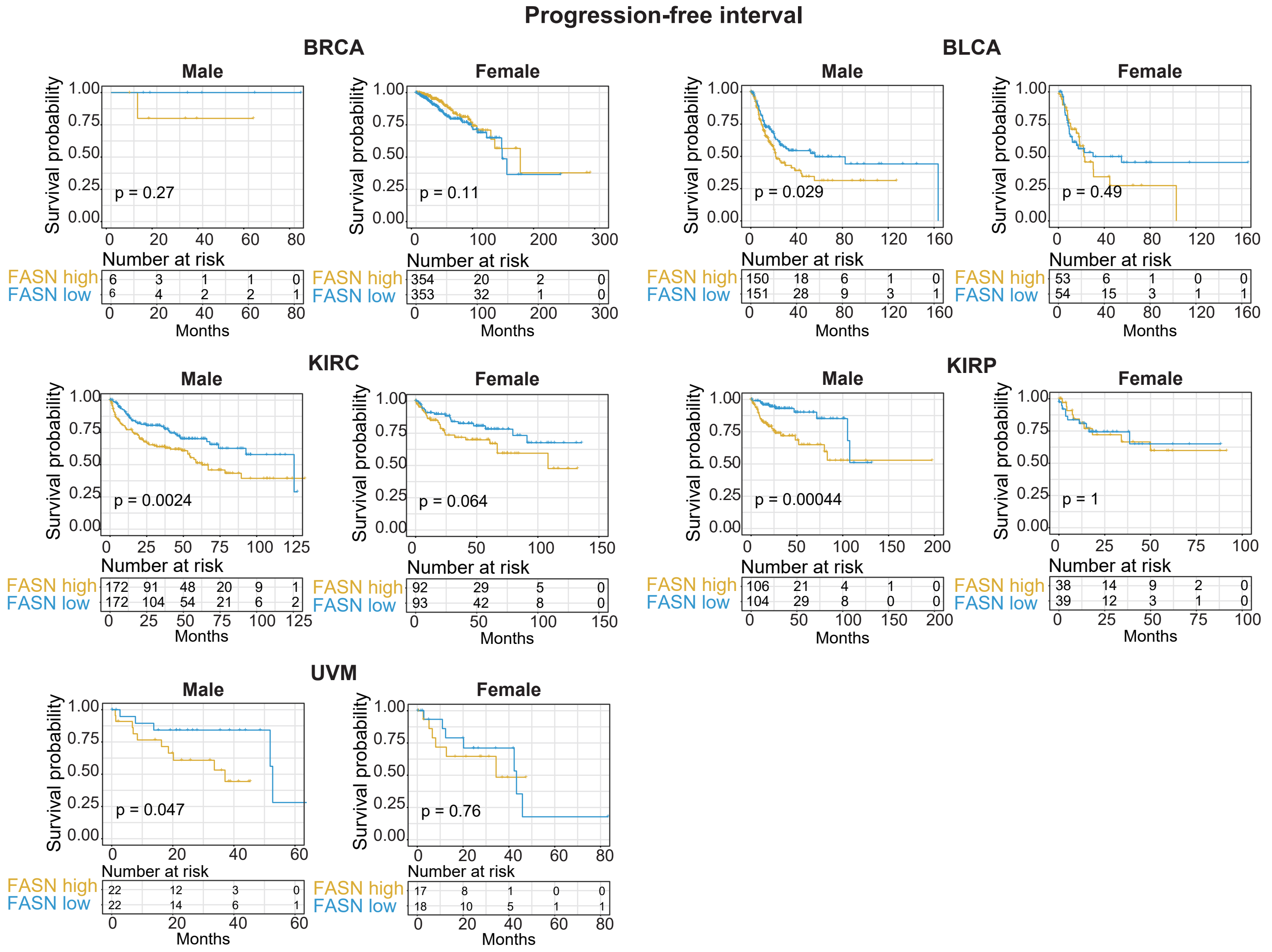

Supplementary Figure 11. Progression-free interval analysis of male and female patients in various cancer types based on the *FASN* expression. Patients are categorized into *FASN*-high (yellow) and *FASN*-low (blue) groups for each dataset according to the median of *FASN* expression. The significance was evaluated by the log-rank test. BRCA: Breast invasive carcinoma; BLCA: Bladder Urothelial Carcinoma; KIRC: Kidney renal clear cell carcinoma; KIRP: Kidney renal papillary cell carcinoma; UVM: Uveal Melanoma.

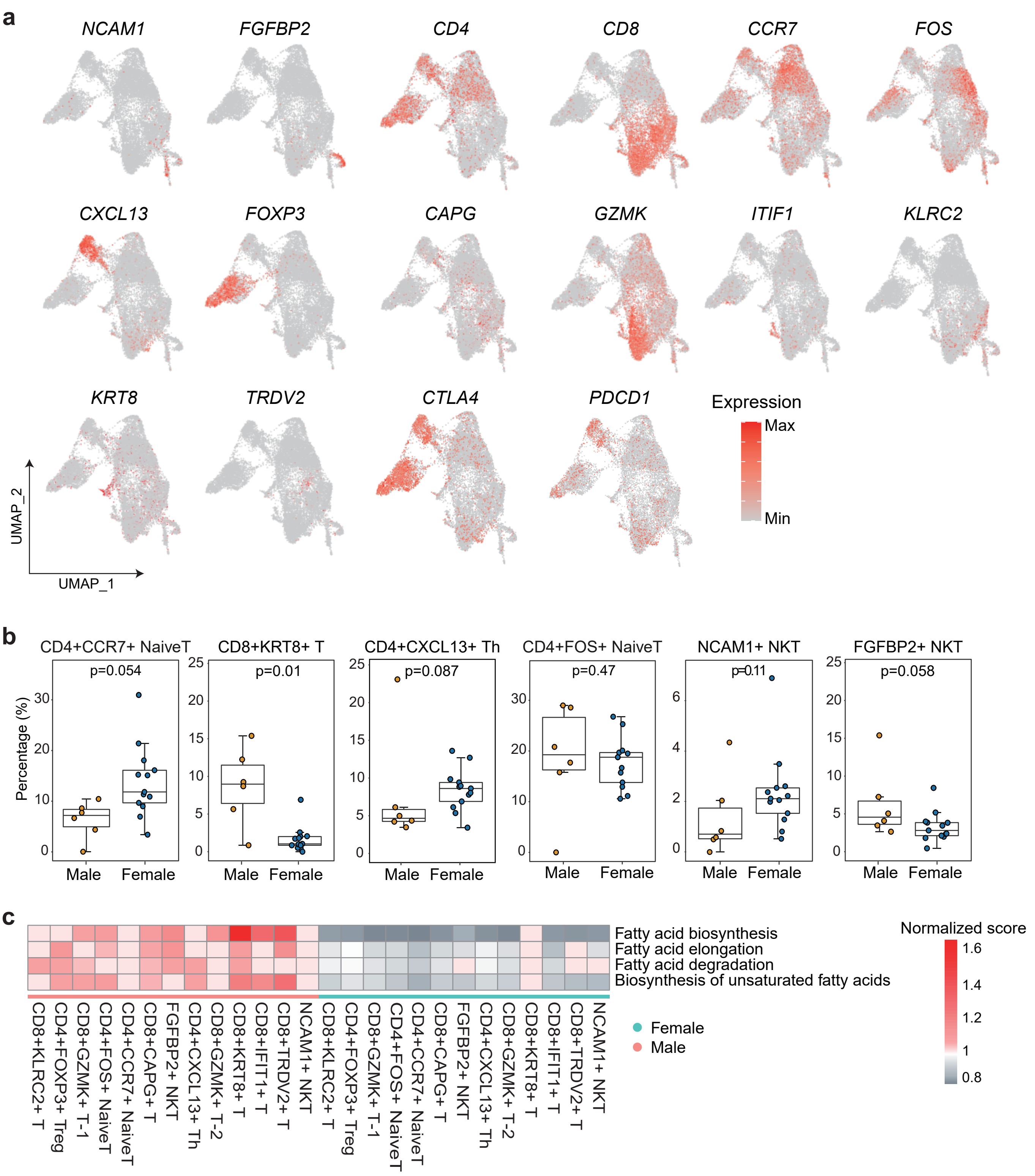

Supplementary Figure 12. Identification and comparison of T cell subpopulations in MBC and FBC samples. a Expression levels of representative genes in each subpopulation (grey to red). **b** Boxplot showing the percentage of *CD4+CCR7+* Naïve T, *CD4+FOS+* Naïve T, *CD8+KRT8+* T, *NCAM1+* NKT, *CD4+CXCL13+* Th, and *FGFBP2+* NKT cells in MBC (n = 6) and FBC (n = 13) samples. P-value was calculated by two-sided Wilcoxon test. **c** Heatmap showing the activity scores of fatty acid metabolism pathways in T cell subpopulations from MBC (lightcoral) and FBC (turquoise) samples. Scale bar depicting low score in grey and high score in red. In (**b**), box plots show median (center line), the upper and lower quantiles (box), and the range of the data (whiskers). Source data are provided as a Source Data file.

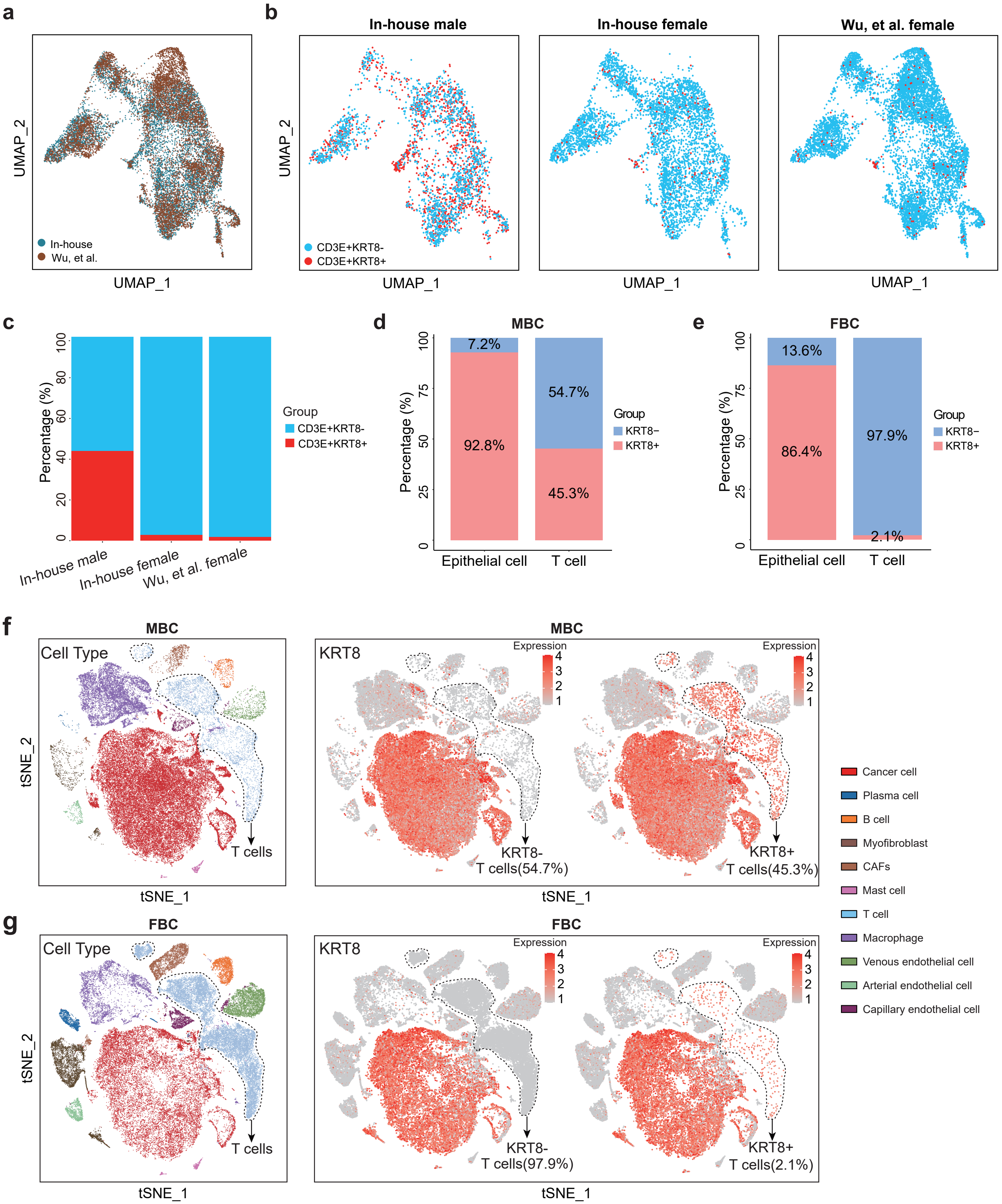
a
b
In-house male
In-house female
Wu, et al. female
UMAP_2
UMAP_1
In-house
Wu, et al.
CD3E+KRT8-
CD3E+KRT8+
c
d
MBC
e
FBC
Percentage (%)
Group
CD3E+KRT8-
CD3E+KRT8+
In-house male
In-house female
Wu, et al. female
7.2%
92.8%
54.7%
45.3%
KRT8−
KRT8+
Epithelial cell
T cell
13.6%
86.4%
97.9%
2.1%
f
MBC
Cell Type
KRT8
Expression
T cells
KRT8-
T cells(54.7%)
KRT8+
T cells(45.3%)
tSNE_2
tSNE_1
g
FBC
KRT8-
T cells(97.9%)
KRT8+
T cells(2.1%)
Cancer cell
Plasma cell
B cell
Myofibroblast
CAFs
Mast cell
T cell
Macrophage
Venous endothelial cell
Arterial endothelial cell
Capillary endothelial cell

Supplementary Figure 13. Evaluation of the existence of *CD3E+KRT8+* T cells in the scRNA-seq dataset. a UMAP plot of T cells colored by data sources. Blue represents in-house samples and brown represents FBC samples from Wu et al. **b** UMAP plots showing the distribution of *CD3E+KRT8-* and *CD3E+KRT8+* T cells in in-house MBC samples (left), in-house FBC samples (middle), and FBC samples from Wu et al. (right). Red represents *CD3E+KRT8+* T cells and blue represents *CD3E+KRT8-* T cells. **c** Barplot showing the percentage of *CD3+KRT8+* T cells in different datasets. Red represents *CD3E+KRT8+* T cells and blue represents *CD3E+KRT8-* T cells. **d** Barplot showing the percentage of *KRT8+* epithelial cells and T cells in MBC samples. Pink represents *KRT8+* epithelial cells and skyblue represents *KRT8-* epithelial cells. **e** Barplot showing the percentage of *KRT8+* epithelial cells and T cells in FBC samples. Pink represents *KRT8+* epithelial cells and skyblue represents *KRT8-* epithelial cells. **f** T-SNE plots showing the cell types and *KRT8* expression in MBC samples. T cells were circled with dashed lines. The feature-plots were split into two separate parts based on whether *KRT8* was positive in T cells. **g** T-SNE plots showing the cell types and *KRT8* expression in FBC samples. T cells were circled with dashed lines. The feature-plots were split into two separate parts based on whether *KRT8* was positive in T cells. Source data are provided as a Source Data file.

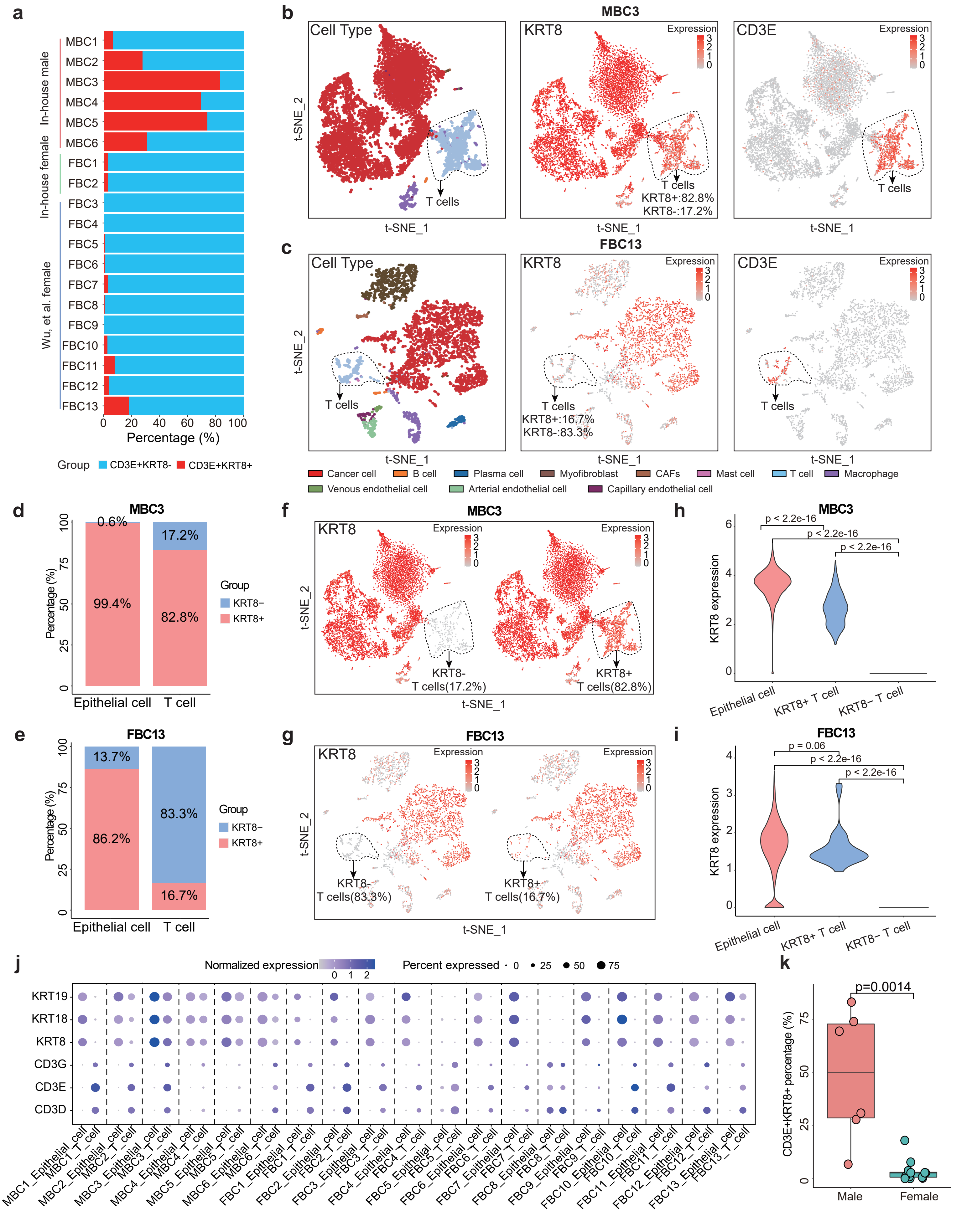

a
In-house male
In-house female
Wu, et al. female
MBC1
MBC2
MBC3
MBC4
MBC5
MBC6
FBC1
FBC2
FBC3
FBC4
FBC5
FBC6
FBC7
FBC8
FBC9
FBC10
FBC11
FBC12
FBC13
Percentage (%)
Group CD3E+KRT8- CD3E+KRT8+
b
MBC3
Cell Type
KRT8
CD3E
Expression
T cells
KRT8+:82.8%
KRT8-:17.2%
t-SNE_1
t-SNE_2
c
FBC13
T cells
KRT8+:16.7%
KRT8-:83.3%
Cancer cell
B cell
Plasma cell
Myofibroblast
CAFs
Mast cell
T cell
Macrophage
Venous endothelial cell
Arterial endothelial cell
Capillary endothelial cell
d
MBC3
0.6%
17.2%
99.4%
82.8%
Percentage (%)
Group
KRT8−
KRT8+
Epithelial cell
T cell
e
FBC13
13.7%
83.3%
86.2%
16.7%
f
KRT8- T cells(17.2%)
KRT8+ T cells(82.8%)
g
KRT8- T cells(83.3%)
KRT8+ T cells(16.7%)
h
MBC3
p < 2.2e-16
KRT8 expression
Epithelial cell
KRT8+ T cell
KRT8− T cell
i
FBC13
p = 0.06
p < 2.2e-16
j
Normalized expression
Percent expressed
KRT19
KRT18
KRT8
CD3G
CD3E
CD3D
k
p=0.0014
CD3E+KRT8+ percentage (%)
Male
Female

Supplementary Figure 14. Evaluation of the existence of *CD3E+KRT8+* T cells in each MBC and FBC sample. a Barplot showing the percentage of *CD3+KRT8+* T cells in each MBC and FBC sample. Red represents *CD3E+KRT8+* T cells and blue represents *CD3E+KRT8-* T cells. **b-c** T-SNE plots showing the cell types, and expression of *KRT8* and *CD3E* in MBC3 (**b**) and FBC13 (**c**). T cells were circled with dashed lines. **d-e** Barplot showing the percentage of *KRT8+* epithelial cells and T cells in MBC3 (**d**) and FBC13 (**e**). Pink represents *KRT8+* epithelial cells and skyblue represents *KRT8-* epithelial cells. **f-g** T-SNE plots showing the *KRT8* expression (grey to red) of epithelial cells, *KRT8+* T cells, and *KRT8-* T cells in MBC3 (**f**) and FBC13 (**g**). T cells were circled with dashed lines. The feature-plots were split into two separate parts based on whether *KRT8* was positive in T cells. **h-i** Violin plots showing the expression of KRT8 among epithelial cells, *KRT8+* T cells and *KRT8-* T cells in MBC3 (**h**) and FBC13 (**i**). P-value was calculated by two-sided Wilcoxon test. **j** Dotplot depicting expression of *KRT8/KRT18/KRT19* and *CD3D/CD3E/CD3G* in epithelial and T cells from MBC and FBC samples. **k** Boxplot comparing the percentage of *CD3E+KRT8+* T cells between MBC (n =6) and FBC (n = 13) samples. P-value was calculated by two-sided Wilcoxon rank-sum test. Box plots show median (center line), the upper and lower quantiles (box), and the range of the data (whiskers). Source data are provided as a Source Data file.

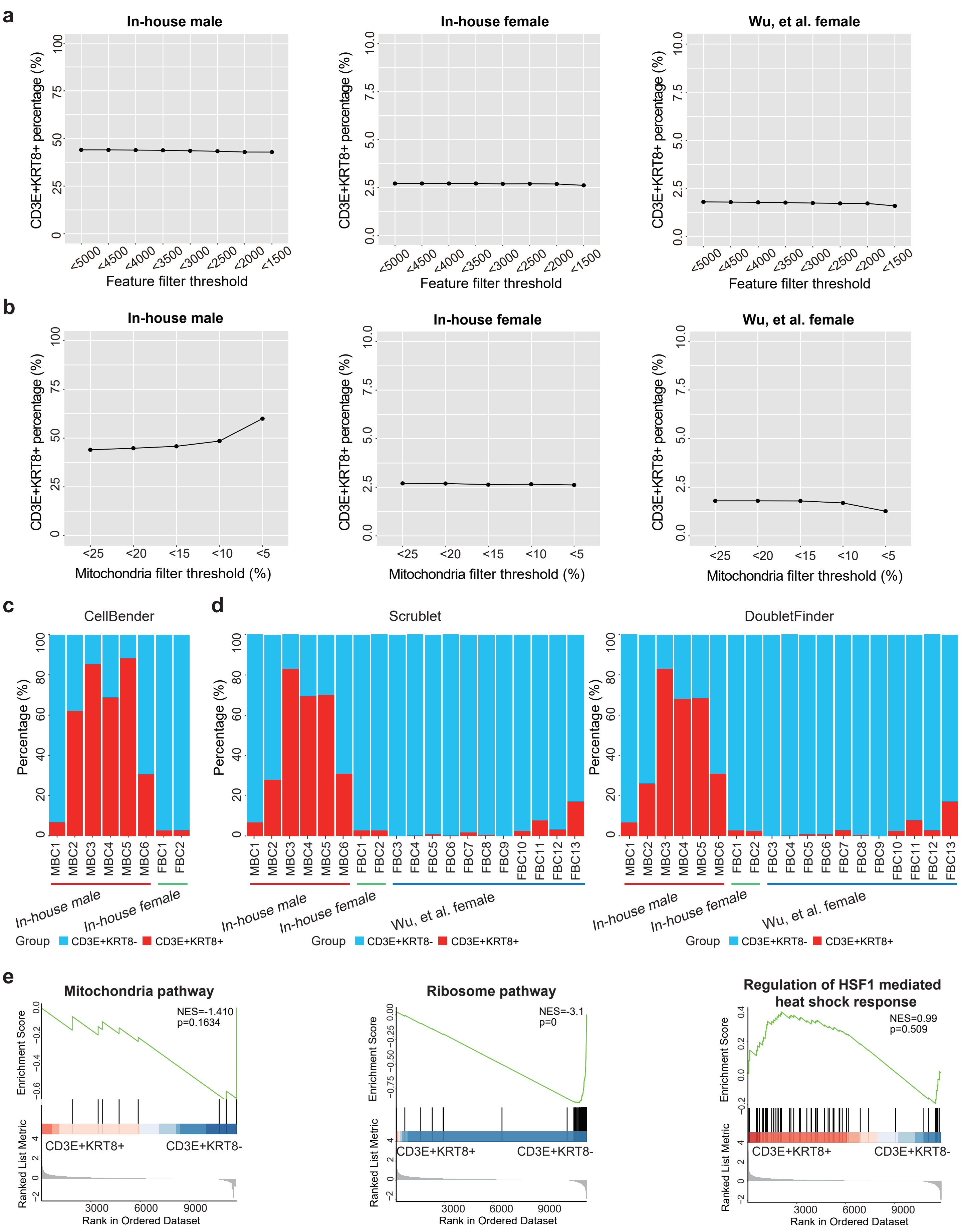

a
In-house male
In-house female
Wu, et al. female
CD3E+KRT8+ percentage (%)
Feature filter threshold
<5000 <4500 <4000 <3500 <3000 <2500 <2000 <1500
b
In-house male
In-house female
Wu, et al. female
CD3E+KRT8+ percentage (%)
Mitochondria filter threshold (%)
<25 <20 <15 <10 <5
c
CellBender
d
Scrublet
DoubletFinder
Percentage (%)
MBC1 MBC2 MBC3 MBC4 MBC5 MBC6 FBC1 FBC2
FBC3 FBC4 FBC5 FBC6 FBC7 FBC8 FBC9 FBC10 FBC11 FBC12 FBC13
In-house male
In-house female
Wu, et al. female
Group CD3E+KRT8- CD3E+KRT8+
e
Mitochondria pathway
NES=-1.410
p=0.1634
Ribosome pathway
NES=-3.1
p=0
Regulation of HSF1 mediated heat shock response
NES=0.99
p=0.509
Enrichment Score
Ranked List Metric
CD3E+KRT8+
CD3E+KRT8-
Rank in Ordered Dataset

Supplementary Figure 15. Evaluating the *CD3E+KRT8+* T cell percentage under different cell-filtering criteria to exclude the influence of low-quality cells. a The line chart showing the percentage of *CD3E+KRT8+* T cells under different feature filter thresholds in in-house MBC samples (left), in-house FBC samples (middle), and FBC samples from Wu et al. (right). **b** The line chart showing the percentage of *CD3E+KRT8+* T cells under different mitochondria filter thresholds in in-house MBC samples (left), in-house FBC samples (middle), and FBC samples from Wu et al. (right). **c** Barplot showing the percentage of *CD3E+KRT8+* T cells in each MBC and FBC sample after removing empty droplets by CellBender. Red represents *CD3E+KRT8+* T cells and blue represents *CD3E+KRT8-* T cells. **d** Barplot showing the percentage of *CD3E+KRT8+* T cells in each MBC and FBC sample after removing doublets by Scrublet and DoubletFinder. Red represents *CD3E+KRT8+* T cells and blue represents *CD3E+KRT8-* T cells. **e** GSEA analysis of mitochondria (left), ribosome (middle) and regulation of HSF-1 mediated heat shock response (right) pathway between *CD3E+KRT8+* and *CD3E+KRT8-* T cells. Two-sided kolmogorov–smirnov test was used to calculate p-value. Source data are provided as a Source Data file.

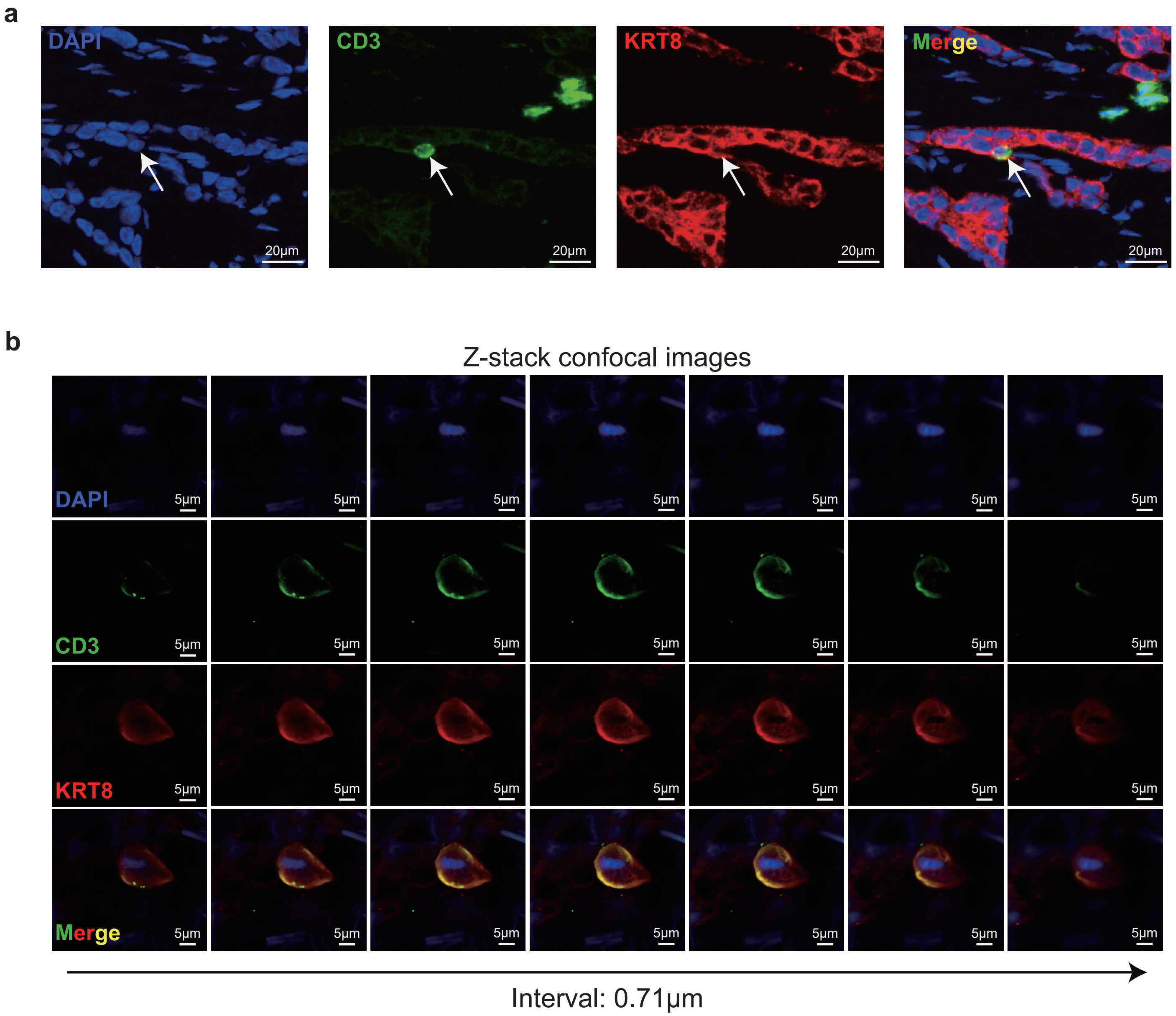

Supplementary Figure 16. Validation of *CD3+KRT8+* T cells using immunofluorescence staining. **a** The immunofluorescence staining of KRT8 and CD3 in an MBC sample. White arrow indicates the CD3+KRT8+ T cell. Scale bar, 20 μm. Experiments were conducted in triplicate. **b** Z-stack confocal images of one CD3+KRT8+ T cell from an MBC sample. Scale bar, 5 μm. The interval for Z-stack was 0.71 μm. Experiments were conducted in triplicate.

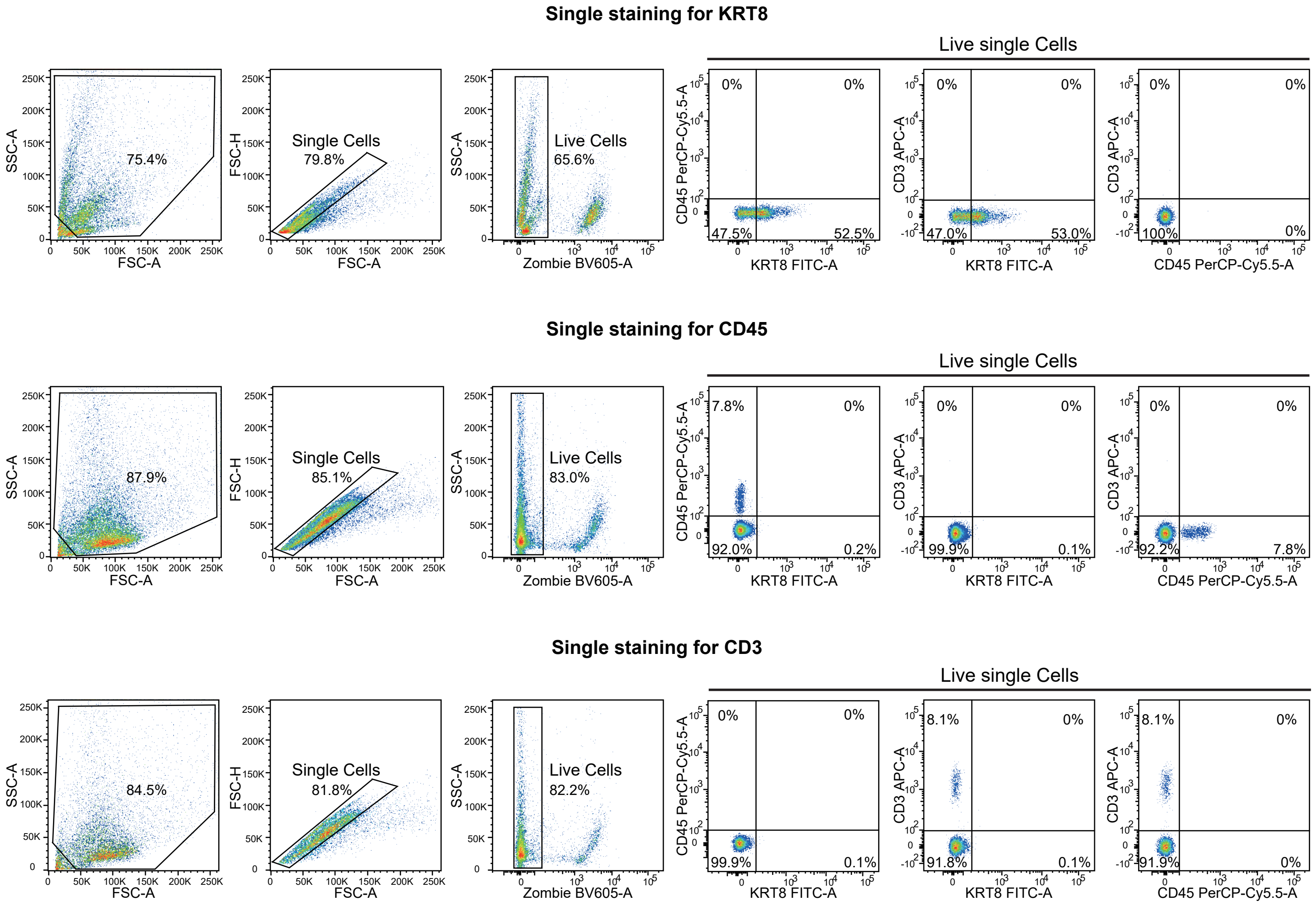

Supplementary Figure 17. Single antibody-labeled compensation controls of flow cytometry analysis for KRT8, CD45, and CD3.

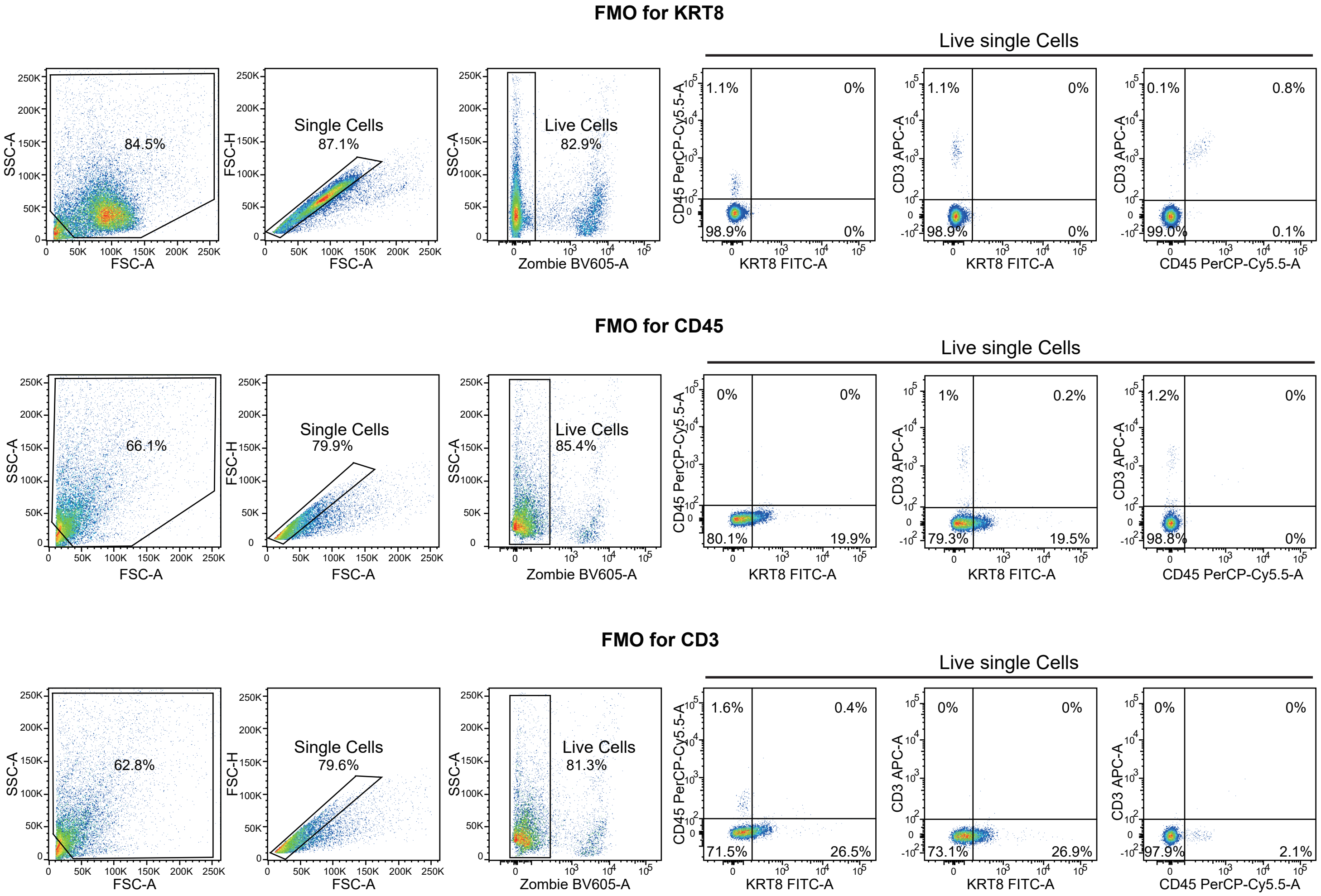

Supplementary Figure 18. Fluorescence minus one (FMO) controls flow cytometry analysis for KRT8, CD45, and CD3.

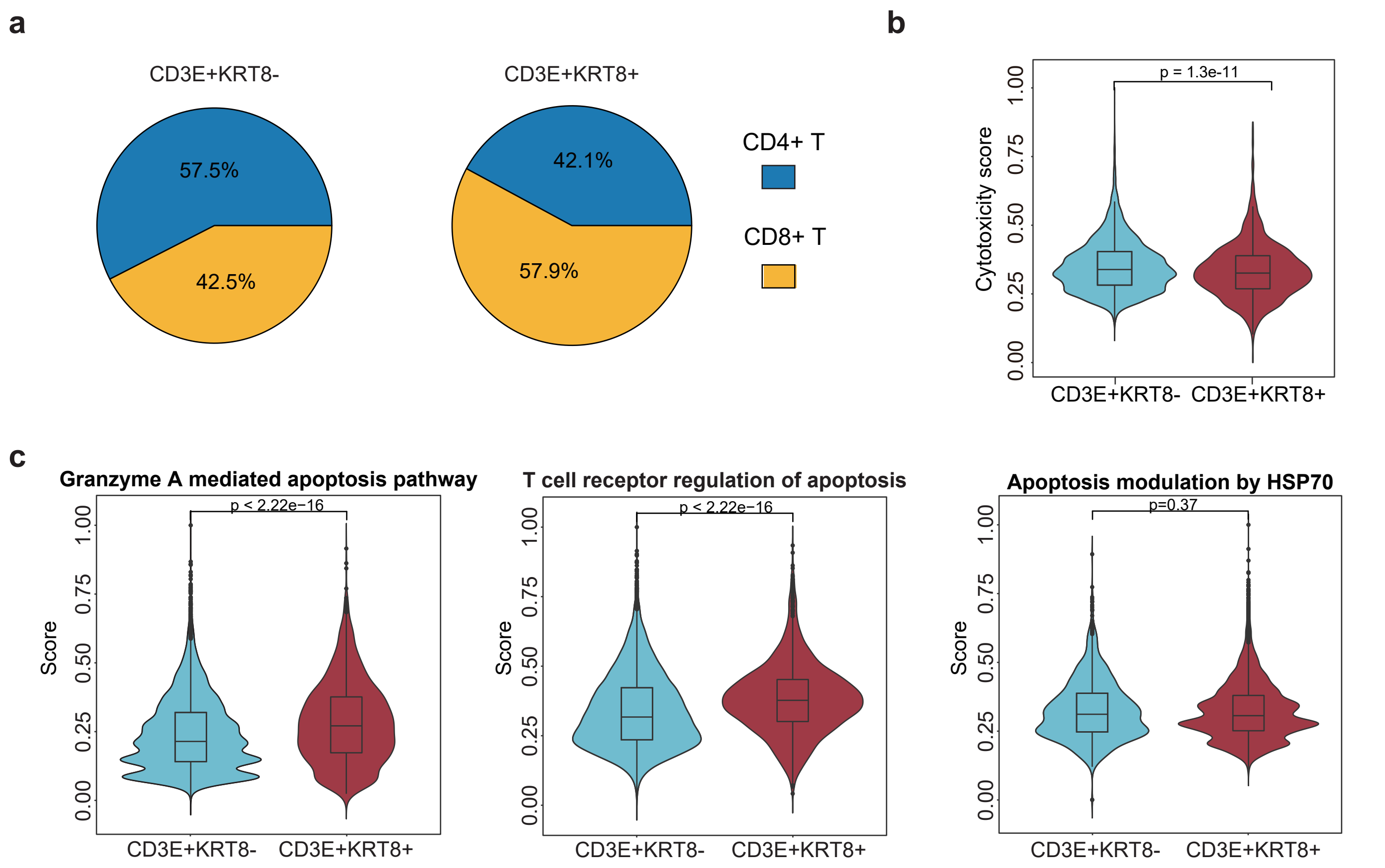

Supplementary Figure 19. Functional analysis of *CD3E+KRT8+* T cells. a Pie charts showing the percentage of *CD4+* and *CD8+* cells in *CD3E+KRT8-* and *CD3E+KRT8+* T cells. **b** Violin plot showing the cytotoxic score between *CD3E+KRT8-* (n = 5983 cells) and *CD3E+KRT8+* (n = 929 cells) CD8+ T cells. P-value was calculated by two-sided Wilcoxon rank-sum test. **c** Violin plots showing the scores of apoptosis-related pathways in *CD3E+KRT8+* (n = 14085 cells) and *CD3E+KRT8-* (n = 1605 cells) T cells. P-value was calculated by two-sided Wilcoxon rank-sum test. In (**b-c**), box plots show median (center line), the upper and lower quantiles (box), and the range of the data (whiskers). Source data are provided as a Source Data file.
