# Supplementary material for: Single-cell transcriptome analysis indicates fatty acid metabolism-mediated metastasis and immunosuppression in male breast cancer
Source: Nat Commun. 2023 Sep 11;14:5590. doi: 10.1038/s41467-023-41318-2 (PMC10495415; doi:10.1038/s41467-023-41318-2)
Supplement: Supplementary file 3 — Description of Additional Supplementary Files [file 41467_2023_41318_MOESM3_ESM.pdf]

Description of Additional Supplementary Files

Title: Supplementary Data 1

Description: Clinical characteristics of MBC and FBC patients.

Title: Supplementary Data 2

Description: Statistical analysis of clinical information.

Title: Supplementary Data 3

Description: The summary of cell number in each sample.

Title: Supplementary Data 4

Description: Gene signatures related to cancer metastasis.

Title: Supplementary Data 5

Description: The metabolic scores of each cancer cell cluster

Title: Supplementary Data 6

Description: The list of up-regulated genes of each T cell subtype.

Title: Supplementary Data 7

Description: The list of specifically expressed genes of CD8+ T cells from male and female samples.

Title: Supplementary Data 8

Description: The list of specifically expressed genes of CD4+ T cells from male and female samples.

Title: Supplementary Data 9

Description: The list of specifically expressed genes of NKT cells from male and female samples.

Title: Supplementary Data 10

Description: The differential expression genes between CD3E+KRT8+ T cells and CD3E+KRT8- T cells.

Title: Supplementary Data 11

Description: Gene signature related to cytotoxic T cells.

Title: Supplementary Data 12

Description: Antibodies for IHC/IF staining and Flow cytometry.
